# The Metaverse as a virtual form of data-driven smart cities: the ethics of the hyper-connectivity, datafication, algorithmization, and platformization of urban society

**DOI:** 10.1007/s43762-022-00050-1

**Published:** 2022-07-28

**Authors:** Simon Elias Bibri, Zaheer Allam

**Affiliations:** 1 Department of Computer Science, Norwegian University of Science and Technology, Sem Saelands veie 9, NO–7491, Trondheim, Norway; 2 Department of Architecture and Planning, Norwegian University of Science and Technology, Alfred Getz vei 3, Sentralbygg 1, 5th floor, NO–7491, Trondheim, Norway; 3 Chaire Entrepreneuriat Territoire Innovation (ETI), IAE Paris—Sorbonne Business School, Université Paris Panthéon-Sorbonne, 75013 Paris, France; 4 Live+Smart Research Lab, School of Architecture and Built Environment, Deakin University, Geelong, VIC, 3220 Australia

**Keywords:** Metaverse, Data-driven smart cities, COVID-19 pandemic, Privacy, Dataveillance, Geosurveillance, Surveillance capitalism, Datafication, Algorithmization, Echo chambers

## Abstract

Recent advances in computing and immersive technologies have provided Meta (formerly Facebook) with the opportunity to leapfrog or expedite its way of thinking and devising a global computing platform called the “Metaverse”. This hypothetical 3D network of virtual spaces is increasingly shaping alternatives to the imaginaries of data-driven smart cities, as it represents ways of living in virtually inhabitable cities. At the heart of the Metaverse is a computational understanding of human users’ cognition, emotion, motivation, and behavior that reduces the experience of everyday life to logic and calculative rules and procedures. This implies that human users become more knowable and manageable and their behavior more predictable and controllable, thereby serving as passive data points feeding the AI and analytics system that they have no interchange with or influence on. This paper examines the forms, practices, and ethics of the Metaverse as a virtual form of data-driven smart cities, paying particular attention to: privacy, surveillance capitalism, dataveillance, geosurveillance, human health and wellness, and collective and cognitive echo-chambers. Achieving this aim will provide the answer to the main research question driving this study: What ethical implications will the Metaverse have on the experience of everyday life in post-pandemic urban society? In terms of methodology, this paper deploys a thorough review of the current status of the Metaverse, urban informatics, urban science, and data-driven smart cities literature, as well as trends, research, and developments. We argue that the Metaverse will do more harm than good to human users due to the massive misuse of the hyper-connectivity, datafication, algorithmization, and platformization underlying the associated global architecture of computer mediation. It follows that the Metaverse needs to be re-cast in ways that re-orientate in how users are conceived; recognize their human characteristics; and take into account the moral values and principles designed to realize the benefits of socially disruptive technologies while mitigating their pernicious effects. This paper contributes to the academic debates in the emerging field of data-driven smart urbanism by highlighting the ethical implications posed by the Metaverse as speculative fiction that illustrates the concerns raised by the pervasive and massive use of advanced technologies in data-driven smart cities. In doing so, it seeks to aid policy-makers in better understanding the pitfalls of the Metaverse and their repercussions upon the wellbeing of human users and the core values of urban society. It also stimulates prospective research and further critical perspectives on this timely topic.

## Introduction

There is much enthusiasm currently about the possibilities created by the Metaverse due the rising prospect that it will greatly impact urban society over the next decade. Touted as the Future of the Internet, the Metaverse has been made possible by the rapid pace of progress in the development of the core enabling technologies, notably Artificial Intelligence (AI), Big Data, the Internet Things (IoT), Edge Computing, Blockchain, Digital Twins (DT), Virtual Reality (VR), Augmented Reality (AR), Mixed Reality (MR), and high-speed 5G networks. While these technologies are not of equal importance in terms of enabling the Metaverse as a *“*sophisticated” computing platform, their convergence has expedited the connection of the existing virtual environments owned by many different platform companies into a 3D network of virtual worlds. Also, the worldwide prevalence and adoption of social media platforms owned by Meta and other big tech companies has significantly facilitated the promotion of the idea of the Metaverse. Meta describes the Metaverse as “a set of virtual spaces where you can create and explore with other people who are not in the same physical space as you. You will be able to hang out with friends, work, play, learn, shop, create, and more” (Bosworth and Clegg 2021). This hypothetical 3D network of virtual worlds combined in an immersive, persistent, transcendent, concurrent, shared cyberspace incarnates ways of living in virtual cities, a form of urbanism in the sense of “the distinctive features of the experience of everyday life in cities” (Bridge, 2009, p. 106), which are being highly responsive to a form of data-driven smart urbanism based on AI and analytics systems. In studying the effects of the emergence of virtual cities have on their perceptions compared to real-world cities, Hemmati (2022) found that the Metaverse can create more believable images than reality.

Research and development of the Metaverse has recently become a key trend in smart urbanism in terms of the design of virtually inhabitable cities based on large-scale data-driven AI systems (Bibri, 2022). Most of the technologies underlying the ecosystem application of the Metaverse (see Lee et al. 2021) are associated with the “horizontal information platform” underlying data-driven smart cities (e.g., Bibri & Krogstie, 2020a; Nikitin et al. 2016), which serves to link together diverse smart technologies and solutions to coordinate urban systems and connect citizens, places, and things. The Metaverse vision depicts the peculiar characteristics of everyday life in data-driven smart cities of the future. While the relationship between virtuality and urbanity has recently become a topic of importance as to how they interact with and impact each other in the digital world, this peculiarity is most likely to affect urban ways of living and urban culture as a product of the perceptual interactions between people and spatial forms. The Metaverse vision points towards shaping “brand new, digitally powered environments [which] can too easily lead to limitations in how the social milieu is framed within them and exclude or render invisible specific social groups, cultures, practices of inhabitation, and places” (Aurigi 2022, p. 2). With reference to smart cities, social exclusion issues include the distortion of the “reality of a city” and the particularities of localities, such as the history, feelings, concerns, knowledge, and trajectories of urban communities (McFarlane and Söderström 2017). Rather, smart cities need to be, as argued by Kitchin (2016, p.11), “framed as fluid, open, complex, multi–level, contingent, and relational systems that are full of culture, politics, competing interests, and wicked problems and often unfold in unpredictable ways.” The Metaverse reduces this complexity into AI-based models and bounded and manageable digital platforms and then employs the outcomes to steer and control citizens in reductionist, linear, mechanical ways. This implies that—as with smart urbanism—there is lack of consideration of the experience of everyday life because of confining urban ways of living to the administrative boundaries of city systems (Verrest & Pfeffer, 2019). In smart urbanism,“both cities and citizens become functional datasets to be managed and manipulated” in order to control urban governance and urban ways of living (Marvin, Luque-Ayala and McFarlane 2016, p.425). Likewise, as noted by Aurigi (2022), much of the rhetoric around the Metaverse—a combination of digital twin visions of “Mirror Worlds” (Gelernter, 1992), pollution and constraint-free living (Benedikt, 1994), and of free-form designs of “Liquid Architectures” (Novak, 1994) as |control-freak utopias echoes the “anti-urban, cyberspace-hailing hype” that emerged in the 1990s. Still, speculative fiction and utopianism play an important role in shaping alternatives to the imaginaries of smart cities (Bina et al., 2020). Indeed, while the Metaverse may seem futuristic, it is edging closer to reality while paving the way for the emergence of virtual cities. As a utopia, the governance of smart cities has been criticized due to the fact that it is strongly driven by government policies and the interests and agendas of big tech companies and large corporations (e.g., Grossi & Pianezzi, 2017; Hollands, 2015). 

Furthermore, the Metaverse was launched amid the COVID-19 pandemic, a crisis purported to be a gigantic opportunity that should be seized to reimagine and reset the world—though mainly in regard to its digital incarnation. And what this entails in terms of both cementing and normalizing the corporate-led, top-down, technocratic, tech-mediated, algorithmic mode of governance, as well as new forms of controlling ways of living in urban society. Since the height of the COVID-19 period, the world has braced for the “new normal” where the use of digital technologies have become mainstream and more embedded into almost every realm of urban society. This “new normal” has already set the stage for unilaterally reimagining and undemocratically resetting the world, resulting in an abrupt large-scale digital transformation of urban society, a process of digitization and digitalization that is in turn paving the way for a new era of virtual reality in future cities (Bibri and Allam 2022). The latter requires the intensification of the datafication, algorithmization, and platformization of both socializing, interacting, working, learning, playing, travelling, shopping, and so on, as well as the whole social organization resulting from these interactions and activities. However, these digital and computing processes are, either intentionally or unintentionally, associated with highly corrosive consequences for urban society (Calvo, 2020). Their outcome epitomizes the core of the Metaverse vision in terms of its ultimate goal to virtualize ways of living and working. Since the onset of this crisis and its multifarious consequences have made it clear that its impact will not fade any time soon, and it will have a long-lasting impact on urban society and ways of living in it. These will be intimately and permanently interwoven with surveillance and control and data-driven governance. The systems deployed to combat the COVID-19 pandemic will become part of the “new normal” in monitoring and governing societies—and hence will not be turned off after the crisis (Sadowski, 2020). Therefore, it has become of crucial importance to understand and find ways to address the risks and impacts of the rapid rollout of technologies across every sphere of urban society on privacy, safety, governmentality, social sorting, and social exclusion, but to name a few (e.g., Aouragh, Pritchard and Snelting 2020 Kitchin, 2020; McDonald, 2020; Stanley & Granick, 2020; Tan, Taeihagh and Tripathi 2021), These concerns are expected to be exacerbated with the Metaverse due to the global architecture of computer mediation upon which the implicit logic of surveillance capitalism depends, and which is constituted by control, commodification, and commoditization mechanisms (Bibri and Allam 2022) that “effectively exile people from their own behavior while producing new markets of behavioral prediction and modification” (Zuboff 2015). Especially, the magnitude of the data to be generated by the Metaverse will be far greater than that being collected from the Internet today due to the immersive nature of VR/AR/MR technologies, adding to the critical questions being raised concerning how Meta and other big tech companies will use these data and for what purposes. This holds true in light of the negative impacts that the social media platforms have had on urban society since the early 2000s. Given the far-reaching implications of the Metaverse, it is timely to critically engage with how, for whom, and with what consequences the 3D network of virtual spaces is emerging within different social settings in which human social interactions take place within different urban contexts.

This paper examines the forms, practices, and ethics of the Metaverse as a virtual form of data-driven smart cities, paying particular attention to: privacy, surveillance capitalism, dataveillance, geosurveillance, human health and wellness, and collective and cognitive echo-chambers. Achieving this aim will provide the answer to the main research question driving this study: What ethical implications will the Metaverse have on the experience of everyday life in post-pandemic urban society?

This paper is structured as follows: Sect. 2 reviews literature on the Metaverse, urban informatics/urban science, and data-driven smart cities, as well as highlights the key aspects of their interrelationship in relevance to the study. Section 3 delves deeper into the ethical implications of the Metaverse and its forms and practices. This paper ends, in Sect. 4, with discussion and conclusion.

## Literature review

### The Metaverse

While the idea of the Metaverse has been around for three decades as a speculative fiction narrative, it is until recently that it came to the public fore with the rebranding of Facebook into “Meta” and other platform providers, gaining increased attention and recognition worldwide. The idea originates from the cult science fiction novel named *Snow Crash,* written by the novelist Neal Stephenson in 1992 (Stephenson, 2003), in which the Metaverse is described as a virtual world that enables users to interact through digital avatars like in the physical world. In the early 1990s, futurists took the idea at face value, incarnating users as avatars in unconnected virtual spaces, and hence found it difficult to connect them into one cyberspace. This has more recently gotten off the ground due to the hyper-connectivity, datafication, algorithmization, and platformization of urban society, coupled with the youthful enthusiasm around interconnectedness: how more people and things can be closely connected*.* However, the idea of people seeing and interacting with each other's avatars is what still makes the Metaverse a speculative fiction rather than a technological vision or a socio-technical imaginary.

**Fig. 1:**
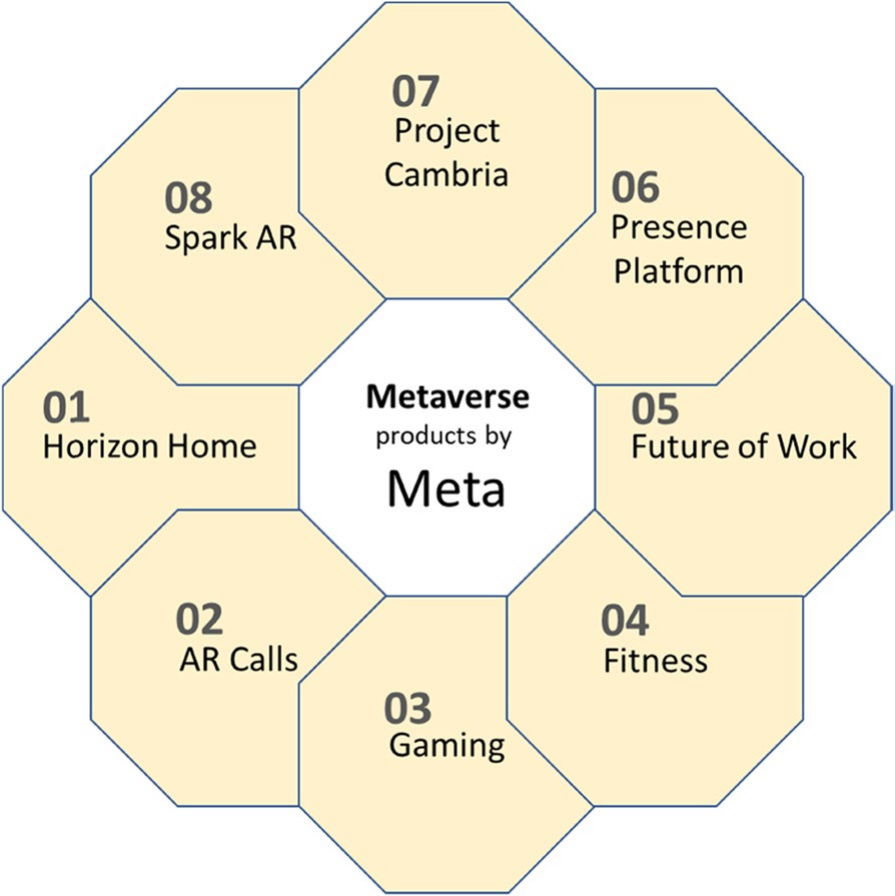
Products announced by Meta during its product launch

As with all new concepts, there is no universal definition of the Metaverse. So, the concept has been defined in multiple ways (see, e.g., Allam et al. 2022a; Duan et al. 2021; Lee et al. 2021; Mystakidis 2022). According to Bibri (2022), the Metaverse is an idea of a hypothetical 3D network of virtual worlds portrayed by its originators as a perpetual, concurrent, transcendent, immersive, and empyrean cyberspace where human users feel tangibly connected to everyday objects and to their real lives, bodies, and minds in the form of avatars with multiple identities and characters. Stephenson’s novel includes a number of concepts and ideas, including VR “headsets” and AR “googles” that allow people to immerse into a fictitious pre-virtual space. The Metaverse has widened the scope of VR and AR to include social interaction, workplaces, shops, leisure, entertainment, creativity, and more, providing a multiuser platform for unlimited interconnected virtual communities and environments using VR headsets, AR goggles, contact lenses, tabletops, hand-held touchscreen devices, and other forms of digital mediation. MR is seen as an enhanced version of VR or a combination of VR and AR, and these three concepts fall under the umbrella term of XR. Lee et al. (2021) offer a comprehensive state-of-the-art review of the Metaverse with respect to the core technologies that fuel the “Digital Big Bang” from the Internet and XR to the Metaverse, which support its ecosystem as a gigantic application. This review includes a detailed account of the immersive technologies underlying the Metaverse. Moreover,  a number of products (Fig. 1) have been initially geared to slowly mould users ’ perceptions towards the Metaverse, including Horizon Home, Future of Work, AR Calls, Gaming, Spark AR, Presence Platform, Fitness, Project Cambria, and more yet to come as the concept becomes universally accepted (see Allam et al., 2022a for a detailed account and discussion in relation to future smart cities) and the platform evolves.

However, Project Cambria is expected to yield high-end VR devices that will encompass the latest technologies. This involves capabilities that are not possible with the currently available VR headsets and virtual avatars (Bonifacic 2021) in order to allow people to interact more naturally in the virtual world and experience and view objects in the physical world in a more realistic way. To reiterate, the Metaverse combines a number of techno-utopian digital visions that emerged in the 1990s, such as “Mirror Worlds” (Gelernter, 1992), pollution and constraint-free living (Benedikt, 1994), and free-form designs of “Liquid Architectures” (Novak, 1994). City governments in Western and Asian societies have, over the last two decades, made efforts for developing and implementing smart cities, and are increasingly engaging in the creation of virtual cities in collaboration with the Metaverse. The Metaverse is seen as a new ecosystem for multifarious interactions and activities across many sectors (see, e.g., Allam et al., 2022a; Lee et al. 2021), thereby becoming a new target for smart cities across the globe to attain new goals through drastic shifts in governance processes. 

### Urban informatics/urban science

As a form of data-driven smart urbanism, the Metaverse involves several interdisciplinary theories and insights from urban informatics and urban science which both entail a computational understanding of city systems and citizens. Urban informatics is concerned with “the study, design, and practice of urban experiences across different urban contexts that are created by new opportunities of real–time, ubiquitous technology, and the augmentation that mediates the physical and digital layers of people networks and urban infrastructures” (Foth et al., 2011). It investigates humans in their interaction with computer and information systems, or of people creating, applying, and using technology and data, in urban environments. As such, it draws on three broad domains: people, place, and technology (Foth et al., 2011). People from different socio–cultural backgrounds include residents, citizens, and community groups, in addition to the social dimensions of organizations and institutions. Place includes both urban sites, locales and habitats, as well as regions, districts, neighborhoods, public spaces, and other kinds of urban areas. Technology involves various forms of urban computing. Further, urban informatics draws on various urban research domains, including urban sociology, urban studies, urban geography, urban engineering, geo–informatics, computer science, data science, software engineering, human–machine interaction, and cultural and communication studies. In terms of research and applications, the major potential of urban informatics lies in four areas (Thakuriah et al., 2017): (1) improved strategies for dynamic urban resource management; (2) theoretical insights and knowledge discovery of urban patterns and processes; (3) strategies for urban engagement and civic participation; and (4) innovations in urban management, and planning and policy analysis. These are also associated with the Metaverse as a virtual form of data-driven smart cities (e.g., Allam et al., 2022a). Overall, as pointed out by Foth et al. (2011), urban informatics emphasizes the new opportunities (including real–time data) for both citizens and city administrations enabled and afforded by ubiquitous computing, in addition to the convergence of physical and digital aspects of the city. Hence, the area of the Metaverse is informed and sustained by urban informatics.

Apart from adding the arts and social sciences to the interdisciplinary mix, urban informatics denotes big data analytics for efficiency and productivity gains in city contexts (Thrift, 2014). This specialized focus in the field of urban informatics has been referred to “data–driven, networked urbanism” (Kitchin 2015) or urban science (Batty, 2013), “a computational modelling and simulation approach to understanding, explaining and predicting city processes” (Kitchin, 2016, p. 4). The strong recursive relationship between these two fields lies in that the former provides the fundamental ideas and key tools to enact urban analytics and data–driven decision–making, and the latter provides the applied domain and raw material (Kitchin, 2016). Urban science seeks to exploit the development of large-scale computation and the growing abundance of data, and to make sense of cities as they are and change by identifying urban relationships, laws and dynamics, as well as predicting and simulating probable future scenarios under different conditions (Bibri, 2021a). In this regard, the fundamental challenges that urban science, as well as urban informatics, deals with are: (1) how to handle and make sense of billions of observations that are being generated on a dynamic basis (Batty et al., 2012) and (2) how to translate the deep insight derived through analytics into new fundamental knowledge and applied knowledge (Foth, 2009; Ratti & Offenhuber, 2014). The longer legacy of scientific and informatics approaches to urbanism, which provide a bedrock of knowledge, is rooted in quantitative geography, urban modelling and digital mapping and geographic information systems, and in urban cybernetics theory and practice (Kitchin, 2016).

Urban science radically extends quantitative forms of urban studies, blending in data science, social physics, and geo–computation (Batty, 2013), which, combined, have high applicability in the Metaverse with respect to the design of virtually inhabitable cities or digital twins of cities. This pertain to the use of simulation modelling to build new digital environments, which tends to “simplify or eliminate what is not functional to the assumed ‘model’ of reality,” (Aurigi 2022) or to “conceal those urban issues, conflicts, and controversies that cannot be represented by digital models and embedded in data analytics techniques” (Bibri, 2021b). In fact, urban science has been criticized within the social sciences for being reductionist, mechanistic, atomizing, essentialist, deterministic, and parochial, collapsing diverse individuals and complex, multidimensional social structures and relationships to abstract data points and universal formulae and laws (Buttimer, 1976), as well as producing policy interventions that have done much damage to city operations (Flood, 2011). Thus, it is important to recognize the complex, multifaceted, contingent, and relational nature of cities, and that they are full of contestation and wicked problems that are not easily captured or steered, and that urban issues are often best solved through citizen-centred deliberative democracy—rather than technocratic forms of governance.

### Data-driven smart cities: the core driving trends and underlying digital and computing processes

The escalating trends towards digitization, digitalization, hyper-connectivity, datafication, algorithmization, and platformization is part of the unprecedented transformative changes that urban society is currently undergoing in light of both recent advances in science and technology as well as drastic shifts in governance. *Digitization* refers to the process of converting information, or encoding representations of urban actions, into a digital format that can be read, processed, transmitted, stored, and shared by computational systems in the form of a series of zeroes and ones that describe a discrete set of points. *Digitalization* is about the ways in which urban processes are organized through and around digital technologies. *Datafication* refers to the practice of taking a social activity, behavior, or process and turning it into meaningful data (Cukier & Mayer-Schöenberger, 2013). It is a name for processes of transforming social action into quantified data, allowing companies and government agencies to carry out monitoring and predictive analytics in real time of digital citizens via AI algorithms (van Dijck, 2014, 2016). *Algorithmization* is the process of algorithmizing different urban activities and processes by converting their informal description into a set of well-defined instructions that can be used to perform a large-scale computation using mathematical and logical rules and models for calculating specific functions (Bibri, Allam and Krogstie 2022). Algorithms have the capacity to analyze vast troves of data constantly generated on citizens and places using AI techniques to make decisions and predict their impacts. *Platformization* refers to “the penetration of infrastructures, economic processes, and governmental frameworks of digital platforms in different economic sectors and spheres of life, as well as the reorganization of cultural practices and imaginations around these platforms*”* (Poell, Nieborg and van Dijck 2019, p. 1). In this network of agents, information, products, services, resources, and values are exchanged among companies, applications, users, and devices. *Hyper-connectivity* as related to the IoT refers to the connectivity and interaction of everything that exist in digital environments, including systems, devices, objects, things, processes, activities, people, and data. Data-driven smart cities represent an immersion in a process of digitization and digitalization enabled by the convergence of the IoT, Big Data, and AI and related infrastructures, and its far-reaching consequences—digital instrumentation, digital hyper-connectivity, datafication, algorithmization, and platformization. These are also at the core of the global architecture of computer mediation pertaining to the Metaverse as a virtual form of data-driven smart cities. 

Smart cities are technologically advanced cities that are able to monitor and understand their environment and citizens and to explore and analyze their processes and actions, respectively, to generate knowledge in the form of applied intelligence that can immediately be used to solve different problems or make changes to improve the quality of life and the health of the city. Data-driven smart cities (e.g., Bibri & Krogstie, 2020b; Dornhöfer et al., 2019; Kaluarachchi, 2022; Sarker et al., 2020; Sutherland & Cook, 2017) are massively digitally instrumented, ubiquitously networked and hyperconnected, intensively datafied, and increasingly algorithmized and platformized, and as such, they enable data-intensive computation across various urban domains based on more innovative techniques, models, and decision systems in the form of large-scale data-driven AI systems in order to enhance and optimize urban operations, functions, designs, strategies, and policies. However, data-driven smart cities are associated with serious risks and hidden pitfalls (e.g., Bibri, 2021c, 2021d; Datta, 2015; Kitchin, 2014, 2016; Luque-Ayala & Marvin, 2015; Marvin et al., 2016; McFarlane and Söderström 2017; Söderström et al., 2014). While data-driven smart cities have provided many new opportunities for transforming urban services and changing the principles of how urban environments can be managed and steered, they have also been driven by other economic and political motives with harmful consequences for citizens. Data-driven smart city systems “become a digital marketplace where citizen-consumers' participation is increasingly involuntary and…are defined through a digital consumer experience that has inherent biases and leaves parts of the city and its population unaccounted for. This renders the city less resilient in the face of future social…risks” (Viitanen & Kingston, 2015). In a similar vein, as a combination of several control-freak and utopian urban visions (Aurigi 2022), the Metaverse will be a digital marketplace where the supremacy and dominance of big tech companies will be further inflated, and the cyberspace will be defined through the experience of human users in the virtual world that will reinforce their control and enslavement—through a prison without walls and from which it is difficult to escape. This renders the Metaverse way less equitable, inclusive, democratic, and safe in the face of future uncertainties and vulnerabilities (e.g., Bibri and Allam 2022; Bibri, Allam and Krogstie 2022; Gurov & Konkova, 2022; Rosenberg, 2022). Nevertheless, digital and computing technologies have shown great potential to improve sustainability, efficiency, equity, safety, resilience, and the quality of life in the context of smart sustainable urbanism (e.g., Bibri 2021e, 2021f), so have immersive technologies to have beneficial psychological effects and to enhance wellbeing with reference to ecological virtual urbanism (e.g., Browning et al. 2020, de Kort et al. 2006, Gerber et al. 2017, Pasca et al. 2021, Yeo et al. 2020). In a nutshell, focusing on advanced digital, computing, and immersive technologies in the context of smart cities in the post-pandemic era may mean losing in human and ethical dimensions (Allam, 2020, Allam and Dhunny, 2019).

## The ethical implications of the Metaverse in post-pandemic urban society

As a way to anticipate the common issues and potential risks, the Metaverse claims to work with experts in government, industry and academia to think through these issues and risks, as well as with human and civil rights communities to ensure that technologies are built in ways that are inclusive and empowering (Bosworth and Clegg 2021). The few areas concerned in this regard are:

-
  - Privacy—how to minimize the amount of data used, enable privacy-protective data uses, and give people transparency and control over their data
  - Safety and integrity—how to keep people safe online and give them tools to take action or get help when they are not comfortable with something they see
-
  - Equity and inclusion—how to ensure technologies are designed inclusively and in ways that are accessible

These recurrent themes remain unilateral claims, which are common to all preceding technological visions or consumer technologies. These indeed emphasize citizen/user progressive participation, wellness and the quality of life, safety and security, support for human social interactions, and provision of new and more efficient services. Both experience and research have shown that big tech companies have failed to live up to such claims as made in the past as part of the vision building process. This leads to the question of why and how the Metaverse would be any different. It is no exception. While this can, to some extent, be a pessimistic viewpoint, it is to be noted that technological innovations are being spearheaded by the same big tech companies that have often been criticized for pushing products and services counter to ethical, human, and social considerations.

### Privacy facets, domains, and harms

Privacy is expected to continue to be one of the major ethical issues that needs to be addressed and overcome when it comes to the use of new technologies. Not only the issue of privacy, but also the issues of security, trust, and accountability have long been a subject of much debate and an area of intensive research in relation to the preceding technological visions, such as Ambient Intelligence (AmI) and Ubiquitous Computing (UbiComp) (e.g., Bibri, 2015; Punie 2003; Stajano & Anderson, 2002; Friedewald et al., 2007). These two metaphors were also “used to depict visions of a future filled with smart, interacting, and interconnected everyday objects and a whole range of immense opportunities and fascinating possibilities…such future will bring that are created by the incorporation of ICT intelligence into people’s everyday lives” (Bibri, 2015, p. 217). However, based on recent statistics published by Johnson (2022), among the concerns posed by the Metaverse according to adults in the USA as of December 2021 are:

-
  - Concern about privacy if Meta succeeds in creating the Metaverse—87%
-
  - Fear that it will be too easy for hackers to impersonate others—50%
-
  - Lack of trust that their identity will be legally protected—47%
-
  - Fear that even more data can be collected and used against them—45%
-
  - Concern of not being sure of the identity of others—43%
-
  - Difficulty in protecting their identity—41%
-
  - Fear that their transactions will not be secure—37

Still, privacy threats should worry the users of the Metaverse most, especially the privacy–enhancing mechanisms proposed thus far remain inadequate to solve this special conundrum. In reality, technology can only safeguard privacy, and even this potential is associated with inherent limitations. Privacy is a real challenge and quandary facing the Metaverse (e.g., Dick 2020, Leenes 2007; Lee et al. 2021; Falchuk et al. 2018). Privacy once considered a basic human right in many jurisdictions and philosophies continues to be falsely enshrined in national, supra-national, and international laws, as it is no longer preserved in a form that ensures it will be safeguarded and respected. This is a real dilemma, irrespective of whether privacy varies between cultures and contexts as a legal concept. Where the realm of privacy actually lies “is a matter of policy, law, and ultimately social norms” (Punie 2003, p. 27). Ongoing debates involve acceptable practices in regard to accessing and disclosing personal and sensitive information about people. Regardless, the era of Big Data marks the end of privacy. Datafication and privacy are strongly interrelated, and this is at the core of the Metaverse given the colossal amount of data that will be collected, analyzed, classified, commoditized, and commodified. For companies, the value of data is not in its presence, but the ways it can be connected to databases and analytic tools (Zuboff, 2019). We are currently experiencing an unprecedented intensification of datafication and algorithmization of smart cities, manifested in the form of new networked, digital technologies permeating the very fabric of everyday life. This poses serious concerns for privacy encroachments. Calvo (2020) address the moral implications of the datafication and algorithmization of urban society within the ethical realm of smart cities.

The user information can relate to various facets and domains creating a number of interrelated privacy forms, including (Martínez-Ballesté, Pérez-Martínez and Solanas 2013; Santucci, 2013):

-
  - Identity privacy—personal and confidential data
-
  - Bodily privacy—the integrity of the physical person
-
  - Territorial privacy—personal space, objects, and property
-
  - Locational and movement privacy—the tracking of spatial behavior
-
  - Communications privacy—the surveillance of conversations and correspondence
-
  - Transactions privacy—searches, purchases, and other exchanges

These forms of privacy will in the realm of the Metaverse be extended and threatened and breached through a variety of unacceptable practices associated with information collection, information processing, information dissemination, and invasion. Each of these causes a different form of harm to people. Privacy breaches include surveillance and interrogation; aggregation, identification, insecurity, secondary use and exclusion; confidentiality, disclosure, exposure, blackmail, appropriation and distortion; and intrusion and decisional interference (Kitchin, 2016), respectively. All harms will raise significant challenges to privacy protection mechanisms in the Metaverse in terms of laws and information practice principles as a question of fairness. Unfairness is a critical issue with respect to the guidelines and principles that will be applied in the Metaverse to behaviorally profile and socially sort users in the virtual world. The Metaverse should take into account the algorithmic fairness as the core value of its designs (Allison et al. 2018) and hence maintain the procedural justices (Lee et al., 2019) to undertake governance roles, which “requires a high degree of transparency to the users and outcome control mechanisms” (Lee et al. 2021). Regardless, algorithmic governance involves unevenness and inequity which reproduce data justice issues (Dencik et al., 2016; Taylor, 2017) across different demographics (Benjamin, 2019; Noble, 2018) with potentially harmful consequences.

The Metaverse will create a number of potential privacy harms for various reasons. These include, but are not limited to, inferencing and predictive privacy harms, dataveillance, geo-veillance, anonymization and re-identification, obfuscation and reduced control, and notice and consent as an empty exercise (see Kitchin, 2016 for a detailed discussion). For example, predictive modelling will be used in the Metaverse to generate inferences about individual users that constitute Personally Identifiable Information (PII), which will be used to access virtual services. Such inferences produce “predictive privacy harms” (Baracos & Nissenbaum, 2014). Tracking data for making inferences which in turn generate inaccurate characterization sticking to individual users, or for sharing personal and sensitive data produced by a predictive model through advertising via the Metaverse, will cause and exacerbate different personal harms. Predicative modelling relates to corporate surveillance where the data collected are more often than not used for commercial purposes and trading with other corporations, as well as regularly shared with government agencies. In this regard, as no data on individual users have been directly collected, the Metaverse will have “no obligation under current privacy regimes to give notice to, or gather consent from its customers in the same way that direct collection protocols require” (Crawford and Schultz 2014, p. 98). Regardless, users must agree with much of what is buried in privacy policy in order to be able to benefit from the so-called virtual services—without reading or understanding what is actually contained in the terms and conditions that they need to check priory to signing in. This is due to several reasons, e.g., they do not have time to read, do not simply care, lack competence in legal matters, or are susceptible to undetectable deceptive methods. The latter relate to the translation of human user experience to surplus behavioral data captured by big data companies in the context of surveillance capitalism. However, it is expected that the deceptive abuses of the Metaverse will be significantly amplified because of the underlying core technologies being specifically designed to fool the senses (Rosenberg, 2022). Irrespective of the means and models to be applied, users will often go with the default—privacy invasion—except if choices are easy, straightforward, and obvious, which will not be the case in the Metaverse. In addition, however, predictive profiles of individual users can be used to socially or politically sort citizens based on certain criteria, assigning a preferential status to certain classes, and marginalizing and excluding other categories. Rather, what is needed is to “design privacy-preserving machine learning to automate the recognition of user privacy preference for dynamic yet diversified contexts in the Metaverse” (Lee et al. 2021, p. 47). However, the difficulty with privacy in the Metaverse is that it is far from clear how and the extent to which the implementation of the principles of the Privacy-by-Design (PbD) approach can be realised. According to Cavoukian (2010), implementing this approach means focusing on, and living up to, these 7 fundamental principles: (1) privacy is proactive—not reactive, (2) privacy as the default setting, (3) privacy embedded into design, (4) full functionality (positive-sum, not zero-sum), (5) end-to-end protection—lifecycle security (6), visibility and transparency, and (7) respect for user privacy (keep it user-centric). It is questionable that Meta will embed privacy in the earliest phased of the development cycle of the Metaverse as a global platform and related products which process personal information. While those principles continue to influence privacy regulation and frameworks around the world, this tends to be largely at the discursive and theoretical levels, particularly in relation to social media platforms. In the post-pandemic era, Meta is occupied with developing strategies for achieving other primary economic. financial, and political goals (see, e.g., Bibri, 2022, Bibri and Allam, 2022, Jonson, 2022, Kasiyanto and Kilinc, 2022, Lee et al., 2021) than devising new approaches to protecting the privacy of individual users and service users.

The issues and risks of privacy invasion—do not seem to be a stumbling block to the social media usage as an everyday activity by the youth age group due to digital illiteracy, psychological manipulation, cognitive dissonance, as well as privacy paradox (i.e., users willingly share their own information). It follows that it is most likely to be the case for the adoption of the Metaverse by the same group. But privacy matters most for the adult group (e.g., Johnson, 2022). One of the foremost problems that the Metaverse needs to solve in order to be socially acceptable is to come up with verifiable privacy mechanisms (Lee et al. 2021). Regardless, the youth group as being more inclined to buy digital products and to extensively use smartphone apps may need to occasionally disconnect for accrued benefits, or, ideally, to wake up to what is being pulled on them as users. Especially, Meta and other big tech companies will continue to ignore the ethics of new technologies by not putting enough efforts to improve existing privacy-protection mechanisms for the benefits of users. They are preoccupied by those technical advancements that only contribute to increasing their control and power, especially data-driven AI techniques. No wonder why the commercial and political value of data has risen astronomically and remarkably, respectively, becoming an unparalleled strategic source of control and power. It is evident that both big tech companies and government agencies hugely benefit from invading privacy under a range of pretexts. Indeed, not only big tech companies, but also governments have been, and will continue to be, the main invaders of privacy (and breachers of security) through platformization under the guise of public health and safety. What is risky to the users of the Metaverse is the idea that this platform will be steered and controlled by big tech companies—considering the aggressive tactics and engagement strategies being currently used in social media platforms for malicious purposes. The risks to the users of the Metaverse cannot be “solved by establishing *strong industry norms* among platform providers or by enacting major changes in *platform business models* from ad-based to subscription-based services.” (Rosenberg, 2022, p. 7), Regarding  public health and safety, it is clear that the solutionist technologies for the COVID-19 pandemic have profound implications for privacy and control creep and reinforce the logic of surveillance capitalism, notwithstanding the reassurance of big tech companies and governments about their effects on civil liberties (Kitchin, 2020).

### Surveillance capitalism

Surveillance is the monitoring or close observation of the behavior and activities of individuals or groups under suspicion—or for no reason. Thus, it could be for the purpose of catching suspects in wrongdoing, information gathering, or of controlling and influencing behaviors. As to wrongdoing, this term could be interpreted based on the official narrative promoted by governments, or be determined by scientifically ungrounded rules and regulations. One example is the behavior of not complying with the draconian measures imposed during the COVID-19 pandemic and reinforced by surveillance technologies, which have proven to be useless in terms of limiting the spread of the so-called virus and to be rather directed towards hidden agenda—mass control. The utility of the solutionist technologies deployed during the COVID-19 pandemic has been oversold, and this crisis “was an opportunity for the state to further roll-out and normalize surveillance technologies and there is little sense that the tracking implemented there will be rolled-back post-crisis” (Kitchin, 2020, p. 371). The systems deployed to combat the COVID-19 pandemic will become part of the “new normal” in monitoring and governing societies—and hence will not be turned off after the crisis (Sadowski, 2020; Stanley & Granick, 2020). Since the onset of this crisis and its multifarious consequences have made it clear that its impact will not fade any time soon, and it will have a long-lasting impact on urban society and the ways of living in it. These will be intimately and permanently interwoven with data-driven governance (e.g., Allam and Jones 2020; Bibri and Allam, 2022). This is a concern of magnitude as the world continues to transition to digital futures. Many citizens—as well as politicians and policymakers—“might believe that surveillance technologies are legitimately deployed if they help to limit the spread of the virus and thereby save lives, regardless of any concerns with respect to privacy or governmentality” (Kitchin, 2020, p. 364). In addition, the same technologies that have demanded fine-grained knowledge about movement, social networks, contact tracing, and health status during the COVID-19 pandemic (Angwin, 2020; Schwartz & Crocker, 2020; Stanley & Granick, 2020) will be utilized by the Metaverse as part of the global architecture of computer mediation upon which the implicit logic of surveillance capitalism depends. The consequences of deploying the surveillance technologies have significant downstream effects that are to be suffered by citizens. Bibri and Allam (2022) explores and questions the Metaverse through the prism of the logic of surveillance capitalism, focusing on how and why the practices of post-pandemic governance are bound to be unethical and undemocratic.

In the era of digitalization, surveillance will be carried out more massively and effortlessly using sophisticated processes of algorithmization and platformzation thanks to datafication and hyper-connectivity. With the Metaverse, the monitoring and surveillance capabilities of new networked, digital technologies will be sharpened and expanded beyond the common methods (e.g., account numbers, credit-card numbers, transaction records, emails, addresses, phone call details, smart card ID) to include smartphone apps, faces, biometric wearables, smart helmets, smart watches, Brain-Computer Interface (BCI), drones, and predictive analytics. As a consequence, the Metaverse will be able to constantly detect and monitor what people do, with whom they talk, where they go, with whom they meet, what they look at, even how long their gaze lingers. Big tech companies have made a science of tracking and characterizing users on their platforms as a result of trading their data (Tucker, 2012) and controlling their behavior. Consequently, it has become very difficult for people to find a place where they can have the right to be left alone and select not to reveal themselves to the world. The use and application of data-driven, compute-intensive, fully automated/autonomous algorithms in “surveillance intelligence” have immensely facilitated the ‘border crossings’ between what is private and public. More than decades ago, Marx (2001) noted that the crossing of four borders usually implies that people feel their privacy is invaded, namely:

-
  - Natural borders such as walls, doors, clothing, phone calls and facial expressions
  - Social borders such as social norms and rules indicating expectations about confidentiality and privacy
  - Spatial and temporal borders such as conveying only different parts of our identity to different people
  - Ephemeral borders such as things or information that go lost or are forgotten.

Not only these borders have been crossed, but also the cognitive and biological ones thanks to the fourth industrial revolution. A number of advanced tools are being deployed that enable new forms of surveillance that run counter to open, free, democratic, and healthy society. The convergence of computing technology and nanobiotechnology makes it nowadays possible to intrude into the hitherto private space of people's minds and bodies, reading their thoughts, manipulating their memories, influencing and modifying their behaviors, suppressing their attitudes, hacking their biological systems, making changes in their genomes (DNA, genes, and chromosomes), and taking decisions out of their hands. This in turn means that users will have to delegate a lot more control and decision power to unpredictable intelligent software agents to have access to virtual services in the Metaverse. For example, the possibilities of decision-making related to data intermediaries in terms of outsourcing human decision points to software intelligent agents acting on individuals’ behalf is being promoted by the World Economic Forum (WEF) as having positive outcomes—despite violating fundamental human rights and civil liberties, The opportunities of autonomous decision-making represent “one of many new policy anchors through and around which individuals may navigate new data ecosystem models. Levers of action for both the public and private sectors are suggested to ensure a future-proof digital policy environment that allows for the seamless…movement of data between people and the technology that serves them” (WEF 2022). What about people’s cognitive ability to act independently? Individuals acquire input from their surroundings and take decisions and actions accordingly while collaborating with institutions and processes as part of self-governance or governmentality.

As the concern about privacy is part of a larger concern about control, about people having control over their own lives, it would be contradictory for the Metaverse to represent their multiple identities, consider their cultural contexts, support their desires and wishes, and respect their freedom and personal autonomy. This is due to the logic of surveillance capitalism that was invented by, or pioneered at, Google in 2001 and later Meta (Zuboff 2016), and that depends “on the global architecture of computer mediation [which] produces a distributed and largely uncontested new expression of power…It is constituted by unexpected and often illegible mechanisms of extraction, commodification, and control that effectively exile persons from their own behavior while producing new markets of behavioral prediction and modification” (Zuboff 2015). The emergence of surveillance capitalism in the wake of the attacks of 9/11 did generate a lot of debate about how these events fundamentally shifted the balance between surveillance and control and privacy and personal autonomy. For example, the control creep that happened post 9/11 was never subsequently rolled back (McDonald, 2020; Sadowski, 2020). Control creep has been occurring since the events of 9/11 across smartphone infrastructures, with technologies designed to deliver specific services being enrolled into policing and security apparatuses (Kitchin, 2020). With the event of the COVID-19 pandemic, technologies beyond smartphone infrastructure, such as the IoT, AI systems, Big Data ecosystems, Edge Computing, XR, Blockchain, are being subject to control creep, i.e., their original purpose is being extended to perform mass surveillance and data-driven governance in order to normalize the new biopolitical architecture of urban society. Central to the biopolitics (Foucault 1977) of the COVID-19 pandemic is “the close management and control of bodies and their circulation and contact; it is thoroughly spatial in its articulation, regulating public and private spaces, spatial access and behaviour, and producing particular spatialities.” (Kitchin, 2020, p. 370) However, it seems that history is about to repeat itself—with regard to the event of the COVID-19 pandemic and its far-reaching and long-term implications for surveillance society. All in all, as a hegemonic discourse, the relationship between the Metaverse, the surveillance capitalism, and the surveillance state is constructed in the light of culturally specific, historically contingent, and episteme conditioned conceptions about the social, political, institutional, economic, and technological changes.

### Dataveillance

As a form of digital surveillance, dataveillance is enacted through the practice of generating, sorting, and sifting datasets in order to identify, monitor, track, regulate, control, predict, and prescribe (Clarke 2017; Raley, 2013). As such, it entails the systematic surveillance of all users' activities and behaviors on the Internet. Monitoring and investigating the digital data pertaining to personal details and online and virtual interactions, actions, and communications will be the primary purpose of the creation and use of data in the Metaverse. This ties in with the process of transforming socializing, working, learning, playing, travelling, shopping, doing business, and so on, as well as the whole social organization resulting from these interactions and activities, into quantified data, allowing Meta to carry out monitoring and predictive analytics in real time of digital citizens/users using AI algorithms, Dataveillance in the Metaverse will intensify or worsen with respect to harvesting and exploiting the data that are collected for other purposes than what users will wish for through connecting numerous virtual settings and services, e.g., to dominate the attention of users more completely and influence their behaviors. Especially, the regulatory frameworks that enable and control dataveillance activities are not—and will ever be—enacted or enforced on big tech companies due to their vested interests with other large corporations and governmental agencies. Data privacy measures and mechanisms have been a subject of much debate since the early 1990s, as well as a great deal of activity in legislatures. This has resulted in, as noted by Clarke and Greenleaf (2017, p. 1) “many countries having data protection oversight agencies and a modest level of jurisprudence. On the other hand, provisions that enable rather than constrain dataveillance are voluminous.” Oversight decisions are largely influenced by the billions of dollars being constantly poured by big tech companies into lobbying while insisting their evolving technology is too complex and fast-moving to be legislated. Regardless, personal data cannot be defined based on privacy regulations alone, as these tend to lag behind technological innovations due to their rapid pace, thereby the need to develop a principled framework that keeps up with them as to what personal data mean. In this respect, Rosenberg (2022) propose some of the regulatory solutions to mitigate the risks of the Metaverse, namely restricting the monitoring of users and their emotional analysis and restricting the virtual product placements and simulated personas within the Metaverse. The author argues that government and industry actors must consider aggressive regulations promptly, predicated on the assumption that it would become difficult to unwind them if the problems are embedded in the business models and digital infrastructure of the Metaverse. Paradoxically, monitoring users and exploiting and monetising their personal data, constitute one of the key elements of the social and economic logic of surveillance capitalism (Bibri and Allam 2022) and thus a prerequisite for its survival as an economic model. In other words, the strength of surveillance capitalism is, paradoxically, also its weakness. Besides, the laws and regulations that many democracies claim to have that seek to restrict the private and governmental use of different forms of surveillance can be argued to be empty signifiers—as clearly exposed during the COVID-19 pandemic. This crisis has exacerbated the issues of the increasing involvement of big tech companies in data policies and data privacy through the accelerated adoption of digital technologies (Li et al. 2022). However, the concept of empty signifier refers to a signifier that is temporarily fixed, and continuously contested and re-articulated, in a political setting determined by power struggles (Laclau and Mouffe 1985/2014). Arguably, the difference between some Western liberal democracies and authoritarian states, which have no or little domestic restrictions, is that the former can now in the COVID-19 era change, reinterpret, or even ignore the established laws—constitution— whenever they wish under the guise of public health and safety concerns and hence emergencies. And then the official narrative gets pushed and advanced by the state-owned and corporate media that are mobilized by the ruling elites. Overall, the missive and pervasive use of digital and computing technologies in data-driven smart cities raises serious governance concerns, which are conveyed or illustrated by the Metaverse as a set of speculative fictional representations—in the form of warning signals and dystopian visions (Bibri 2022). As pointed out by Bina, Inch and Pereira (2020, p. 8), “the dystopian consequences of elite rule through advanced technology and the imposition of a strictly rational and controlled social order are pervasive features of the future urban worlds imagined in fiction. The sacrifice of individual freedoms and privacy to technologies of surveillance is part of the imposition of totalitarian social controls.” 

Our everydayness has been entangled with information collection, information processing, and information dissemination, and our wirelessly connected digital world generates overwhelming amounts of data, compromising our privacy which is expected to be completely eroded in the virtual worlds of the Metaverse. This allows big tech companies to, over sufficiently long periods of time, extract “irreplaceable values” to influence and shape the way people behave in the digital/virtual world in the form of deep insights associated with harmful applications. The world is turning into constellations of instruments across many spatial and temporal scales and morphing into a haze of software instructions, which are essential to the further expansion and power of big tech companies and large corporations. This also serves the voracious appetite of government agencies for mass control and constant surveillance, manifested in dataveillance practices diversifying and proliferating across various spheres of urban society—thanks to recent advances in AI, the IoT, and Big Data technologies and the implications of their convergence: hyper-connectivity, datafication, algorithmization, and platformization.

 With these sophisticated digital and computing processes, users in the Metaverse will be prone to be affected by much greater levels of intensified scrutiny as more and more aspects of their everyday life become quantified and captured as data. Indeed, it will be all but impossible for users in the Metaverse to live their everyday life without leaving traces themselves and other traces captured about them. Digital footprints and shadows (Dodge & Kitchin, 2005) are due to the pervasiveness of digitally mediated and virtual interactions, communications, and activities and hence surveillance and control, adding to the increasing use of unique identifiers to access a myriad of virtual services. The digital experimentation of the Metaverse as a cyberspace may, if realized and widely deployed without societal norms and established legal protocols, become extremely harmful to human rights and take on the appearance of anarchy in the virtual world due to the non-recognition of the systems controlling ethical conduct. The Metaverse will most likely not comply with the national and international law and regulations for protecting fundamental rights as to the dignity of human users, the respect for their moral worth, and the distribution of risks and benefits. Unjustifiably violating people's privacy is a byproduct of surveillance that is too often criticized by civil society organizations and that has been exacerbated by the COVID-19 pandemic. Therefore, it is important to, as stated by (Kitchin, 2020):

-
  - Document the ways in which a new surveillance and biopolitical regime is being produced through the alliance of government control and surveillance capitalism and their use of a range of technologies.
-
  - Examine the application and effects of emerging surveillance regimes on different communities.
-
  - Chart how people are resisting, subverting, and seeking to enact forms of data justice.

### Geosurveillance

The Metaverse is a 3D network of always-on virtual worlds where spatial scales and time scales will completely be collapsed by the use of real-time data. The datasets to be created will show immediately the operation of the real-time cyberspace but also imply how long term changes in the behavior of individual users can be detected and how to deal with them. The Metaverse will also have the possibility to connect and search isolated available databases containing personal and sensitive information. It will have access to all data on individual users at a fine spatial and temporal scale where they can be identified for different purposes—thanks to geosurveillance technologies. Geosurveillance is the tracking and tracing of location and movement of people, vehicles, goods, objects, products, and services and the monitoring of interactions and relationships across space and time. With its partnership with those companies that run geosurveilance and provide operating systems for smartphones (e.g., Google, Hikvision Digital Technology, Dahua Technology, Axis Communication, and Motorola Solutions), as well as with those cyber-intelligence companies offering their services to governments (e.g., Cellebrite, Intellexa, Cobwebs Technologies, Rayzone Group, Verint Systems, and Patternz), the Metaverse will possess a vast quantity of highly detailed spatial behavior data on users.

The widespread diffusion of multiple wireless technologies, especially high-speed 5G networks, will further optimize the real-time sensing and collection of massive repositories of spatiotemporal data that represent society-wide proxies for human interactions, communications, and activities. The rollout of 5G technologies has been accelerated and intensified in the wake of the COVID-19 pandemic. While Meta as well as Google, Apple, and Microsoft are generating real time location and movement data, the COVID-19 pandemic has laid it bare through sharing such data and analytic tools to perform movement monitoring (Kitchin, 2020). Concerning quarantine enforcement/travel permissions, citizens in China are required to install a smartphone app and then scan QR codes when accessing public spaces to verify their permission to enter based on their infection status (Goh, 2020). Taiwan has deployed a mandatory phone-location tracking system to enforce quarantines, issuing fines for violations to those straying beyond their lockdown range (Timberg & Harwell, 2020). To ensure compulsory home quarantine is observed, Hong Kong has issued electronic tracker wristbands (Stanley & Granick, 2020). A number of other governments or authorities in Poland, Germany, Italy, Russia, Israel, South Korea, Singapore, USA, United Arab Emirates, and more have introduced similar apps or other technologies (e.g., smart helmets and biometric wearables) for varied purposes related to quarantines, movement tracing, social distancing, and others (Kitchin, 2020). As a way to help combat the COVID-19 pandemic, a number of companies are actively repurposing their platforms and data. Google and Apple are developing solutions to aid contact tracing via smartphones (Brandom & Robertson, 2020); Google is monitoring the effects of interventionist measures globally; and Meta, Apple, Google, and Microsoft are generating and storing real-time location and movement data while legitimating surveillance capitalism as well as invasively harvesting and exploiting personal (behavioral) data for profit-making (Kitchin, 2020). These pro bono activities enable the COVID-19 washing of surveillance capitalism through the laundering of reputations (McDonald, 2020; Stanley, 2020). As a consequence, the fine-grained mass tracking of movement and proximity will enable tighter forms of control and have frightening effects on democracy and civil liberties (Bibri and Allam 2022). Such practice is legitimized because “authoritarianism—for the ‘right’ reasons—starts looking tolerable, even good, because it looks like the only option” (Sadowski, 2020).

Datasets on human location and movement provide a powerful social microscope aiding in extracting patterns and models and their seamless composition with further analyses. The models and patterns characterising the trajectories people follow during their daily activities across different spatial and temporal scales will be evaluated, and the behavioral exploration of these models and patterns will be performed using visual analytics in order to build simulation and prediction methods for decision making pertaining to the users of the Metaverse. Indeed, from spatial behavior data, lots of other insights can be deduced (e.g., mobility, lifestyle, activity, interests, social category, culture, religion) and also shared with other partners for commercial or governance purposes. The consequence of corporations and institutions generating spatiotemporal data and using analytics to extract insights is that individuals will be tracked and traced at different spatial and temporal resolution scales, and will become open to geo-targeted profiling and social sorting (Kitchin, 2016). The monitoring of movement and location and constructing mobility profiles and histories have become pervasive, dynamic, automatic, autonomous, continuous, and cheap owing to geosurveillance technologies and their infiltration into the very fabric of data-driven smart cities, paving the way for the era of virtual cities and hence the Metaverse. Geosurveillance technologies include, but are not limited to:

-
  - Remote controllable digital Closed-Circuit Television (CCTV) cameras equipped with facial recognition software programs
-
  - Traffic and red-light cameras; congestion and toll cameras
-
  - Active Global Positioning System (GPS)
-
  - Unique ID transponders
-
  - Automatic Number Plate Recognition (ANPR) cameras (a mass surveillance device for optical character recognition)
-
  - Smart card tracking in buildings
-
  - Sensors mounted to bins, lampposts, shops, and malls
-
  - ATMs and other automatic machines in transport stations, hospitals, and others
-
  - Drones
-
  - Smartphones and smartphone apps
-
  - Smart helmets and smart watches

Several companies in the UK and the US have offered their facial recognition services to identify co-proximity patterns in public and private spaces and to link testing results with facial recognition to regulate movement by means of “immunity passports” (Kitchin, 2020; Proctor & Devlin, 2020). A secretive data analytics company (McDonald, 2020; Sadowski, 2020) has offered their services to monitor and model the spread of the COVID-19 pandemic to predict the required response of health services in the UK and other states (Hatmaker, 2020). Meta is sharing movement and location data with researchers to monitor social distancing (Paul et al., 2020). With respect to smartphone apps, for example, there are many iPhone and Android apps that transmit location data to a third party other than the app developers. The bonanza of indexical, real-time location-based data harvested through smartphone apps and recording and transmitting location has significantly grown since the mid-2000s (Angwin 2014; Kitchin, 2020). With the use of geosurveillance technologies, selected citizens will be digitally tagged to enable tracking through various tools and devices that continuously transmit location and status information via a mobile or wireless network to a monitoring system for processing, dissemination, and invasion. Geosurveillance technologies continue to proliferate on a hard-to-imagine scale and to be massively deployed across cities and regions globally, supported by the roll-out of advanced wireless communication networks—5G and 6G. The growing capabilities of 5G amounting to up to 10 Gb/s are providing new opportunities to the Metaverse as a giant ecosystem that relies on the real-time transmission of colossal amounts of data. Increase in connectivity hinged on current 5G speeds and anticipated 6G connectivity speeds is expected to play a significant role in realizing the Metaverse vision. Especially, it is expected that the Metaverse’s requirements will exceed 5G’s available bandwidth (Braud et al., 2020). However, these high-speed network technologies are associated with serious risks to human health (e.g., Allam et al. 2022b). They may disfigure the city landscape to stave off serious health damages by high frequency microwave radiations. In 2017, over 250 doctors and scientists from the European Union (EU) raised concerns about the health hazards associated with 5G and called governments to impose a moratorium on the rollout of these technologies (Cassauwers 2021).

### Human health and wellness

User addiction and the problematic use of the Metaverse are difficult to address and overcome. This is further compounded by the COVID-19 pandemic and its long-term impact on the ways of living and working in cities. Considering the shift prompted by this crisis: from face-to-face meetings or social gatherings to their virtual forms, a large body of recent work has indicated that the prolonged usage of such forms could create other problems in terms of the abusive use or addiction to the Internet (Garcia et al. 2020). According to recent statistics and facts, the dangers of the Metaverse based on the Internet users worldwide in 2021 include addiction to a simulated reality with 47% and mental health issues with 41% (Johnson, 2022). Complete dependence on the Metaverse due to immersive experiences will result in mental and physical health problems, adding to impairment and inaction in users' functions in their everyday life. Addiction to immersive technologies through the excessive use of the Metaverse products and services has generated controversies in scientific, technological, and medical communities. This issue is expected to be the target of further research, debate, and criticism, as well as to be approached from a variety of perspectives. This implies that evidence-based strategies and recommendations for policy makers will be difficult to develop and implement.

According to Meta, in the Metaverse “you will be able to hang out with friends, work, play, learn, shop, create and more. It is not necessarily about spending more time online. It is about making the time you do spend online more meaningful.” This is self-contradictory because the way in which the cyberspace will be designed in terms of the range of activities that can be performed in it is most likely to prompt incessant use of immersive technologies—at least for the youth group of society. In other words, what the Metaverse entails actually reveals the opposite given that it will allow multiple avatars of real people to interact with each other and with aesthetic beautiful objects in a variety of visually appealing virtual worlds, thereby attracting youngsters to spend more time online meaninglessly. VR researchers have studied the relationship between behavioral addictions and virtual environments as well as the underlying causes (Segawa et al. 2019). There are many immersive components that are strongly associated with psychological states that may occur when human interaction with physical environment take place in VR/AR, including peak experiences, optimal attention span restoration, and positive emotions (Bibri 2021g). The everyday life scenarios in VR/AR allow designers to simulate the influence of real-life settings on emotional states. Immersive simulations are likely to increase the restorative effects of projected everyday environments in virtual  spaces. In addition, users could experience super-realism that allows them to experience many activities that resemble the real-world settings. Concerning spending time meaningfully, the Metaverse cloaks the fact that what is meaningful to an individual user or a group of society might not be the case for another, predicated on the assumption that people have subjective interpretations depending on such factors as culture, age, education, and socio-economic status. Overall, it is unknown what users may experience once entered (plugged) in the Metaverse due to the kind of unlimited virtual services they will (be psychologically manipulated to) benefit from. This involves swaying users' emotions to get them to act or feel in a certain way while fully immersed in virtual environments.

The abusive use of the Metaverse will have severe mental and physical harms. These include, over a prolonged period of time, depression, anxiety, social dysfunction, unhappiness, loneliness, aggression, and dissatisfaction with life. These in turn relate to the kind of lifestyles in which little or no physical activity could be performed, which may increase the risk for obesity, diabetics, neurological diseases, cardiovascular diseases, and others. In addition, social technologies cause stress, destroy jobs, and make people more materialistic, which harm their quality of life through more choices and efficiencies (Heitman, 2011), in addition to creating new problems by solving old ones. Furthermore, user safety issues will be prevalent in the Metaverse. The physical harm is a problem with respect to the injuries caused by the use of VR headsets and AR googles.

Although the Metaverse has the potential to evolve into something immersive for certain groups of society, it portrays dystopian images of the future (see Bibri 2022 for a detailed discussion on dystopianism). This is clearly reflected in how it envisages the digital world and configures users and their social interactions, adding to ignoring ethical values (e.g., integrity, truthfulness, faithfulness) and social values (e.g., freedom, respect, responsibility). Digital society does not practice many values, which are unfortunately crucial for social wellbeing in terms of belongingness to and making contributions to communities. VR could lead to the behavior changes of massive users, which could lead to discernible impacts on society (Colley et al. 2017, Tong et al. 2017). The idea that the Metaverse will be in the varied scenarios of people’s everyday lives, causing a drastic transformation to their everyday life, is unfolding as part of science fiction narratives. In this respect, Johnson (2022) illustrates the things that the Metaverse will allow people to do that they would not do in real life, including:

-
  - Alter consciousness with the help of VR
-
  - Create an alter ego of the opposite sex or different age
-
  - Spend a lot of money on collective clothes or accessories
-
  - Play adult games that engage in extreme violence and/or sex
-
  - Conduct unethical experiments on virtual humans
-
  - Watch virtual executions
-
  - Engage in hate speech

These things give some insights into the kind of the extreme behaviors that the Metaverse will encourage or induce certain individual users to engage in that have negative impacts on the guiding values of society. These include positive assertiveness skills, the ability of being authentic to oneself in all situations, engagement with other people in one’s community, treating others with respect, and communicating with others in a direct and honest manner without intentionally hurting their feelings. Cyber-dystopias portray a world with dehumanizing experiences in terms of depriving people of human qualities, such as courage, honesty, kindness, self-awareness, and wholeheartedness. Overall, the Metaverse as a set of highly realistic virtual environments will enable users to try things that are impossible in their real life, e.g., replicating immoral events (e.g., Lewis et al. 2021), with the underlying assumption that it will drive users to extend their usage time and further exacerbate the addiction to the immersive media.

### Collective and cognitive echo chambers

Social media platforms play a key role in facilitating both collective and cognitive echo chambers. A social media echo chamber is when users experience biased, tailored experiences that eliminate differing perspectives and opposing viewpoints thanks to AI algorithms. Also referred to as “filter bubbles” (Pariser, 2011), echo chambers often pertain to the dangers of the rise of social media usage as an everyday activity for billions of people. The effects of echo chambers are expected to intensify in the Metaverse thanks to always-on virtual environments and immersive technologies. The Metaverse will be algorithmically designed in ways that make users, especially the youth group, encounter only views or opinions that coincide with their own, or that induce them to engage with content that confirms their already-held beliefs. This will result in users developing a tunnel vision in relation to their real world and hence easily accepting the prevalent narratives advanced by the Metaverse through finding their beliefs, views, or opinions constantly echoing back to them. This will in turn reinforce their individual belief systems and exclude alternative ideas concerning societal, ethical, and political issues due to the decline of their exposure to other opinions or views. Therefore, with reference to the Metaverse, it is important to promote the fairness of the recommendation systems in order to minimize the biased contents and thus impact the user behaviors and decision making (Steering 2021), as well as to ensure the contents are appropriate to diversified users and to consider personalised content display in front of them (Lee et al. 2021). Still, the rampant censorship of some contents is another major issue that is difficult to overcome in light of the unpredictable and sometimes unjustified regulations adopted by social media platforms. In fact, social media platforms use such regulations in response to what governments dictate in terms of their total control over the narratives advanced by the ruling elites. This practice creates a society that is dependent on the dominant channels of communication and interaction and the hierarchy of the establishment (Bibri 2022). Gillespie (2018) addresses in more detail the current practices of social media platforms and explains the underlying rationales for how, why, and when censorship policies are enforced. In doing so, the author highlights that content moderation received too little publish scrutiny despite its shaping influence on social norms and its consequences for the fabric of society and cultural production. 

However, a number of methods and techniques have, over the past  two decades, been developed, implemented, evaluated, and enhanced for this purpose in social media platforms, especially when it comes to pandemics, economic downturns, financial crises, wars, and others. During the COVID-19 pandemic, the effects of echo chambers have worsened in social media platforms. This has been manifested in people becoming insulated from discussions, debates, and enlightenments; manipulated into sharing misbeliefs and misinformation; engaging with the official narratives promoted by authoritarian states and large corporations; and being lured into interacting within groups holding similar beliefs, thereby the illusion of widespread agreement. However, this doesn’t not necessary mean that echo chambers have no doors. It is rather up to the users of social media platforms to open certain doors and to keep others shut—depending on their needs for information in order to be able to take well-informed behavioral decisions, especially for certain demographics such as education, age, and culture. As individual users within virtual communities “have ties to multiple networks and therefore cannot be said to be structurally confined to an echo chamber, they may nevertheless be confined to a psychological or cognitive echo chamber as they do not pay equal attention to the information brought forward by their ties outside of the secluded echo chamber network” (Schlegel 2019). This relates to confirmation bias in terms of human tendency to disregard information contradicting already-held beliefs and seeking out information confirming these views as a way to avoid cognitive dissonance. This concept denotes inconsistent beliefs and thoughts pertaining to behavioral decisions, manifested in confusion, feeling conflicted over a disputed matter, and being aware of conflicting views and clueless what to do with them.

The cognitive echo chambers facilitated by social media platforms have recently led to immediate repercussions in regard to the non-informed behavioral decisions that a substantial number of people have made in relation to the draconian measures and absurd mandates imposed on them by the governments in several democratic societies. The set of principles aiding people in interpreting their everyday reality have been greatly shaken off due to the rise of virtual networks and communities. A number of spiritual, religious, intellectual, social, and political beliefs have been influenced and shaped due to the wide use of social media platforms in which ideological frames have been amplified and made disproportionally salient. Frames are associated with the selection of some aspects of a perceived reality and rendering them more salient in order to push certain narratives, promote causal interpretation, and to trigger moral evaluation. They are equated to social representations, which are culture–specific and conventionalized by society and attuned to its values, as well as prescriptive in the sense that they represent a force, a combination of structures and traditions that shape the way people think and what they ought to think (Moscovici, 1984). Fisher (1997, p. 5) describes cultural frames as “socio–culturally and cognitively generated patterns which help people to understand their world by shaping other forms of deep structural discourse.” Fundamentally, cultural frames are reconstructed, transformed, or challenged through social interactions, thereby the counterproductive effects of eco-chambers. One level of echo chambers describes a certain network of organizations postulating frames and narratives in line with their ideology or worldview.

In the Metaverse, users are expected to be less and less exposed to diverse perspectives and ideas, and to be inclined to form groups of like-minded avatars framing, reinforcing, and reflecting certain beliefs, views, or opinions, as well as existing feelings or habits. With respect to the former, the issue lies in locking users into perpetual social groups and inflicting tangible damage to their understandings, thereby limiting their freedom to think critically and to learn to tolerate different views. Users are expected to become even polarized and trapped in virtual environments and networks that promote and exchange one-side or extremist content. These may magnify the social impacts of virtual echo chambers and digitally alienating spaces (Evans, 2011; Newton, 2021), or cause to abuse social media engagement strategies to manipulate users with biased content (Shou, 2021), misinformation, or news from incredible sources. Advanced machine and deep learning algorithms (e.g., natural language) will be used in the Metaverse to influence users through periodic, ephemeral, and tailored experiences in ways that eliminate opposing viewpoints. Advanced immersive capabilities will make it even easier to lure people into believing what fits their preferences and hence find themselves in a comfortable, self-confirming feed in a persistent way. This will in turn make it difficult for certain groups of society (especially youngsters) to  understand and recognize the feelings and attitudes of other people, a social skill that can otherwise be improved through person-to-person social interaction in real-world settings. Worth pointing out given the complexity surrounding echo chambers as existing in a collective entity of meaning construction and individual cognition, it will be difficult to develop evidence-based recommendations or to come up with counter measures. It remains insufficient to just change the recommender systems and 3D network of virtual spaces embedded in the design of the Metaverese and the corresponding AI techniques to combat self-produced echo chambers or to mitigate the risks of those that shape and collectively transform individual belief systems.

## Discussion and conclusion

For three decades, the idea of the Metaverse, just like the idea of the smart city for much of the twentieth century, was only a science or speculative fiction that was pictured in the popular media. But quite suddenly with the increasing convergence and massive proliferation of dominating disruptive technologies, the prospect of the parallel post-reality universe may become the new reality, at least for certain groups of society. This always-on virtual environment is quite different from anything that human users have experienced hitherto. While the Metaverse may have the potential to create new experiences through the immersive technologies of VR/AR and unleash unparalleled creativity, it raises critical issues, serious risks, and provocative questions. This justifies the increased scholarly interest in exploring this emerging potentially transformative phenomenon.

The aim of this paper was to examine the forms, practices, and ethics of the Metaverse as a virtual form of data-driven smart cities, paying particular attention to: privacy, surveillance capitalism, dataveillance, geosurveillance, human health and wellness, and collective and cognitive echo-chambers. Achieving this aim in turn provided the answer to the main research question driving this study: What ethical implications will the Metaverse have on ways of living in post-pandemic urban society? The COVID-19 pandemic—has cemented and normalized the hyper-connectivity, datafication, algrithmization, and platformization of urban society, which is argued to be not for the better, as well as compounded by the increasing virtuality of everyday life pushed by the Metaverse. The ethical implications identified include, privacy encroachments, security breaches, behavioral manipulation, mind control, human health decrease, personal data exploitation and commodification, citizen disempowerment, and social sorting due to predictive analytics. While some of these issues and risks are reputedly recognised by big tech companies, they will be exacerbated in the Metaverse due to the massive misuse of the digital and computing processes underlying the associated global architecture of computer mediation. This is leading to a number of critiques concerning the underlying concepts, ethos, forms, and practices of the Metaverse. One response to these critiques is to argue that the Metaverse needs to be re-cast in ways that re-orientate in how users are conceived; recognize their human characteristics; and take into account the moral and social values and principles designed to realize the benefits of socially disruptive technologies while mitigating their pernicious effects.

In particular, rather than being cast as quantifiable, knowable, manageable, and tractable users that can be steered and controlled and thus their behavior can be predicted in mechanical, linear ways, users need to be framed as humans that are self-motivated, self-determined, blessed with conscience, rational and moral beings, having feelings, and called to holiness, and that are fully characteristic of culture, religion, free choices, and behave in spontaneous ways. Reducing this complexity into models and behavioral data and then using the outcomes to control and guide the experience of everyday life produces a reductionist, linear, rationalistic, and limiting understanding of human users, as well as overly technocratic and authoritarian forms of governance. Regardless of its underlying logics and rationales, the Metaverse should not trump the experience of everyday life in driving the governance of urban society towards a dystopian world.

The Metaverse offers a seemingly inspiring vision of the digital future of reality and what can be done to make it happen, and this can possibly be expanded to the creation of a new platform to address the common challenges associated with existing technologies. Yet, this is seldom the focus of new technological visions and the intention of their creators, especially when it comes to the overall balance of human users' moral, psychological, behavioral, intellectual, social, and political wellness. As digital and computing technologies have become more sophisticated and deeply embedded into the fabric of urban society, they are instigating and unleashing far–reaching social transformations—with negative intended and unintended consequences. As there is undoubtedly a dark side to technological development in terms of their negative aspects that are usually kept concealed, whether they are meant to be for our collective disadvantage has already been witnessed and deeply felt during the COVID-19 pandemic and its ramifications on the lives of citizens across the globe. And more of this side is yet to be seen, as the digital world evolves and thus the agendas —with fraudulent labelling— become visible and the claimed sounding noble goals unfold.

The assumption of a better virtual world made in the Metaverse could become an apocalyptic trip of an autonomous repression to normality from inside the human brain and body, without the option of creative explorations and the ability to stop unwelcome changes. Connecting virtual worlds and supplying virtual services in the Metaverse is meant to generate, sort, and sift datasets on human experience to identify, monitor, track, trace, profile, and prescribe people, distort their cognition, dominate their attention, and modify and control their behavior even more completely. Emerging technologies reinforce hierarchies and power imbalance by knowledge concentration, social exclusion, unbalanced distributions of benefits, constant surveillance, unequal power relations, privacy loss, democracy erosion, corporatization governance, anticipatory governance, social sorting, psychological manipulation, and so forth.

The hiding of the Metaverse in daily aesthetically pleasant virtual environments is like the wolf in sheep’s clothing, pretending that this technology is harmless and only designed to provide free virtual services. There are often hidden agenda behind most of technological visions that are painted in sunny colors in that they will bring humans an easier, happier, more efficient, and more pleasant life. The Metaverse will accelerate the inevitability of the use of AI techniques and agents to control, predict, and shape users’ cognition, emotion, motivation, and behavior in the so-called empyrean cyberspace. Sadly, it is almost impossible to make the scientists, engineers, technologists, and corporatists involved in the Metaverse take responsibility for not placing enough importance on and understanding the full extent of the critical issues and risks in question. They have repeatedly rejected the established norms of societal responsibility and accountability —while building empires on the details of our private lives as a new form of exploitation and exceptionalism that seeks to shape, direct, and control our intimate inner lives beyond merely strip-mining them (Zuboff 2019). Ominously, people are providing the new raw material and labour, for free, to big tech companies to cement and normalize their surveillance and thus increase their control, profit, and power, thereby participating fully in the Metaverse and the becoming-virtual of much of the human world at the expense of fusing their bodies and minds more intensively with the cybersphere. Nonetheless, there are lots of stumbling blocks to and grand challenges for the realisation and social acceptance of the Metaverse as a virtual alternate to real-life world beyond the COVID-19 crisis that paved the way for its emergence.

## Data Availability

Not applicable.
